# Evidence Suggesting a Role of Iron in a Mouse Model of Nephrogenic Systemic Fibrosis

**DOI:** 10.1371/journal.pone.0136563

**Published:** 2015-08-25

**Authors:** Chhanda Bose, Judit K. Megyesi, Sudhir V. Shah, Kim M. Hiatt, Kimberly A. Hall, Oleg Karaduta, Sundararaman Swaminathan

**Affiliations:** 1 Central Arkansas Veterans Healthcare System, Renal Section, Medicine Service, Little Rock, Arkansas, United States of America; 2 University of Arkansas for Medical Sciences, Department of Internal Medicine, Division of Nephrology, Little Rock, Arkansas, United States of America; 3 University of Arkansas for Medical Sciences, Department of Dermatology, Little Rock, Arkansas, United States of America; 4 University of Arkansas for Medical Sciences, Department of Pathology, Little Rock, Arkansas, United States of America; RWTH Aachen, GERMANY

## Abstract

Nephrogenic systemic fibrosis is associated with gadolinium contrast exposure in patients with reduced kidney function and carries high morbidity and mortality. We have previously demonstrated that gadolinium contrast agents induce *in vivo* systemic iron mobilization and *in vitro* differentiation of peripheral blood mononuclear cells into ferroportin (iron exporter)-expressing fibrocytic cells. In the present study we examined the role of iron in a mouse model of nephrogenic systemic fibrosis. Chronic kidney disease was induced in 8-week-old male Balb/C mice with a two-step 5/6 nephrectomy surgery. Five groups of mice were studied: control (n = 5), sham surgery control (n = 5), chronic kidney disease control (n = 4), chronic kidney disease injected with 0.5 mmol/kg body weight of Omniscan 3 days per week, for a total of 10 injections (n = 8), and chronic kidney disease with Omniscan plus deferiprone, 125 mg/kg, in drinking water (n = 9). Deferiprone was continued for 16 weeks until the end of the experiment. Mice with chronic kidney disease injected with Omniscan developed skin changes characteristic of nephrogenic systemic fibrosis including hair loss, reddening, ulceration, and skin tightening by 10 to 16 weeks. Histopathological sections demonstrated dermal fibrosis with increased skin thickness (0.25±0.06 mm, sham; 0.34±+0.3 mm, Omniscan-injected). Additionally, we observed an increase in tissue infiltration of ferroportin-expressing, fibrocyte-like cells accompanied by tissue iron accumulation in the skin of the Omniscan-treated mice. The deferiprone-treated group had significantly decreased skin thickness (p<0.05) and significantly decreased dermal fibrosis compared to the Omniscan-only group. In addition, iron chelation prevented tissue infiltration of ferroportin-expressing, fibrocyte-like cells. Our *in vitro* experiments demonstrated that exposure to Omniscan resulted in the release of catalytic iron and this was prevented by the iron chelator deferiprone. Deferiprone inhibited the differentiation of human peripheral blood mononuclear cells into ferroportin-expressing cells by immunohistochemical staining and western blot analysis. Our studies support an important role of iron in the pathophysiology of gadolinium chelate toxicity and nephrogenic systemic fibrosis.

## Introduction

Nephrogenic systemic fibrosis (NSF) is associated with gadolinium contrast use in patients with reduced kidney function [1,2] and carries high morbidity and mortality [3,4]. We have previously demonstrated that gadolinium contrast agents induce systemic iron mobilization [5] and differentiation of peripheral blood mononuclear cells (PBMC) into ferroportin-expressing fibrocytic cells [6]. Further, we have observed that there is increased tissue infiltration of ferroportin-expressing fibrocytic cells accompanied by tissue iron accumulation in NSF [3,6]. Gadolinium salts have been shown to disrupt iron metabolism at a cellular level [7]. Iron is capable of causing tissue injury through its ability to donate electrons and thereby trigger oxidative stress [8,9]. Additionally, iron can induce the transmetallation of gadolinium chelate to liberate “free” gadolinium and thus cause further injury [10]. However, the role of iron in the pathogenesis of NSF has not been examined previously. In this study, we examined the role of iron in gadolinium toxicity utilizing a mouse model of NSF and provide supportive evidence with *in vitro* cell culture models.

## Materials and Methods

Male Balb/C mice 6 to 8 weeks old (Jackson Laboratories, Bar Harbor, ME, USA) were used for the study. Mice were given ad libitum access to water and chow (Harlan Teklad, Madison, WI, USA). The mice were housed at an ambient temperature of 22°C ± 2°C, hygrometry of 45 ± 10%, with 12/12h light/dark cycles, and acclimatized for 1 week before starting the experiments. Mice were maintained in our Veterans Administration Medical Unit (VAMU).

### Ethics Statement

This study was carried out in strict accordance with the recommendations of U.S. National Institutes of Health Guide for the Care and Use of Laboratory Animals. The protocol was approved by the Institutional Animal Care and Use Committee (IACUC approval number: 9-08-2). All surgeries were performed under anesthesia and all efforts were made to minimize pain and suffering.

### CRF Surgery

To establish chronic kidney disease, a 5/6 nephrectomy (5/6 Nx) was performed in two stages under 80 mg/kg sodium pentobarbital anesthesia. Briefly, the left kidney was exposed and decapsulated to avoid ureter and adrenal damage, then the upper and lower poles were partially resected via a left flank incision using an AARON 950 Electrosurgical Generator from BOVIE (St. Petersburg, FL, USA). One week later (week 0), the entire right kidney was removed via a right flank incision. The mortality rate in the first week after right nephrectomy was 10%. In the sham surgery control mice, a left flank incision was performed at week 1, both poles of kidney were identified, then the flank incision was closed; a subsequent right flank incision was performed at week 0, the right renal artery was identified, then the flank incision was sutured.

### Preliminary studies to establish a model of nephrogenic systemic fibrosis in a mouse

**Fig 1:**
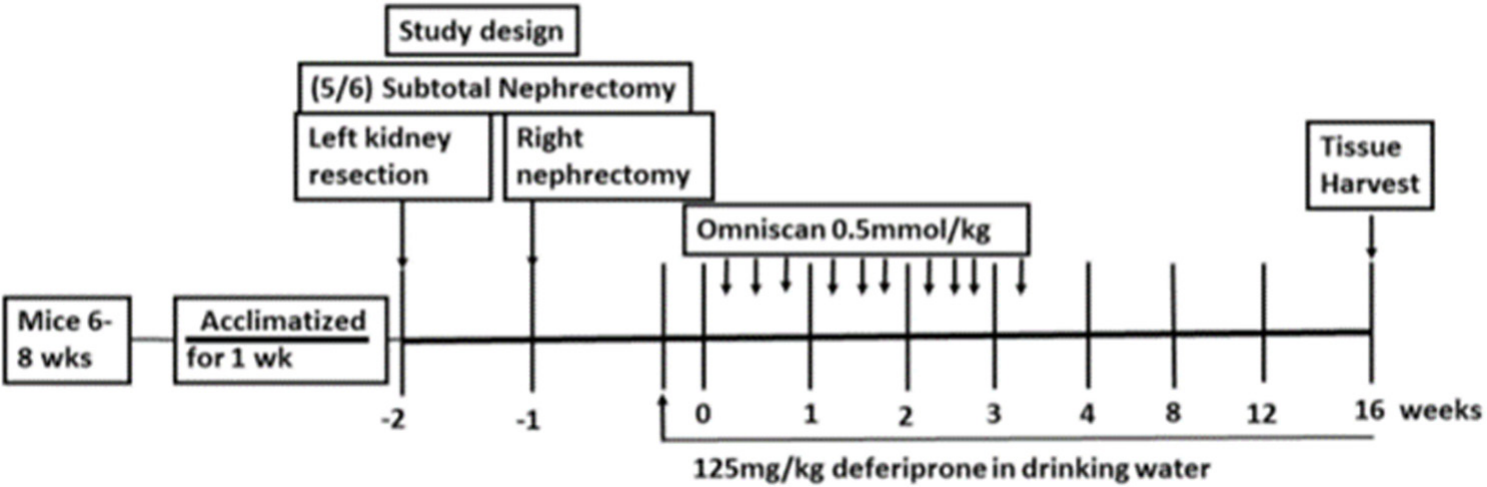
Study design and treatment schedule for Omniscan injections and deferiprone treatment. As mentioned in Materials and Methods, after one week of establishment of CKD, one group of mice was administered deferiprone in drinking water (125mg/kg) two days before starting Omniscan injections. Three injections of Omniscan (0.5 mmol/kg, tail IV, 100 μl volume) per week on alternate days were given to two groups of mice (one received deferiprone and the other received Omniscan only) excluding weekends. A total of 10 injections were given. Control mice (one group of CKD and other was sham) animals received 10 injections of an equivalent volume (100 μl) of saline. After 16 weeks at the endpoint, animals were sacrificed and blood and tissues were collected.

The dose of Omniscan and time were titrated to establish the optimum required to consistently induce NSF. After one week of nephrectomy, CKD mice were divided into 5 groups, i.e. injected with either normal saline (0.9%) or those treated with various doses (0.1, 0.3, 0.5 and 1 mmol/kg body weight) of gadodiamide (Omniscan, GE Healthcare, Princeton, NJ), for three days per week (days 1, 3, 5, days of the week, tail vein, IV, in 100 μl volume) and a total of 10 injections were given. Mice were monitored every day for any skin changes including hair loss, reddening, ulceration, and skin lesions and skin tightening. Dorsal skin biopsies were taken weekly after Omniscan treatment and at sacrifice. Mice that received 1mmol/kg Omniscan developed early tail necrosis from the 2nd week after injections and manifested tail degeneration by 3–4 weeks. Mice that were moribund or showing obvious signs of distress were removed from the study and euthanized. In view of overt early toxicity with 1 mmol/kg dose and recommendations by our animal use committee, we conducted our further experiments in mice treated with 0.1, 0.3, and 0.5 mmol/kg doses of Omniscan and these mice were monitored for longer periods. There were no such overt early toxic skin changes in the mice that received other doses of Omniscan. Dorsal skin biopsies were taken weekly. The samples were analyzed histopathologically and by immunohistochemistry in the dermatopathology lab by a qualified dermatopathologist (KMH) with extensive expertise in reading NSF. Mice treated with lower doses of Omniscan (0.1 and 0.3 mmol/kg bw) developed NSF-like skin changes by 18 to 20 weeks; in comparison, mice treated with 0.5 mmol/kg bw developed skin changes suggestive of NSF by 10 to 16 weeks. Mice treated with 0.5 mmol/kg bw started developing changes in skin from the 10th week (presence of CD34+ and procollagen+ positive and fibroblast-like cells). By the 16th week more clinically visible changes of NSF were evident including hair loss, indurations, and skin tightening. Skin biopsy samples showed a significant increase in CD34, CD163^+^ dermal spindle cells, and thick collagen bundles with skin thickness, as determined by immunohistochemical and histopathological analysis. Thus, for our intervention studies we used 10 injections of 0.5 mmol/kg Omniscan, three injections per week, for three consecutive weeks. Mice were sacrificed 13 weeks after last injection. The total duration of the study was 16 weeks after the first Omniscan injection (Fig 1).

### Omniscan injection and treatment with iron chelator

Five groups of mice were studied: Group 1—control without CKD; Group 2—sham surgery (no CKD); Group 3—CKD control; Group 4—CKD with Omniscan; Group 5—CKD with Omniscan and deferiprone. Based on preliminary studies, Groups 4 and 5 mice were injected with 10 injections of 0.5 mmol/kg body wt. for three days per week (days 1, 3, 5, days of the week), tail vein, IV, in 100 μl volume). Groups 2 and 3 were injected with100 μl normal saline (0.9%) at the same time points as above. Deferiprone was administered to Group 5 in drinking water mice two days before Omniscan injection started, as shown in Fig 1. Deferiprone treatment (125 mg/kg body weight) [11,12] was continued until the termination of the experiment. All groups had free access of chow diet (Harlan Teklad) and water. Water consumption and food intake were registered biweekly. Mice were sacrificed 13 weeks after last injection (Fig 1). The total duration of the study was 16 weeks after the first Omniscan injection.

### Macroscopic skin findings and histology

The mice were examined daily for macroscopic skin changes such as fur loss, reddening, ulcerative lesions, scab formation, and skin tightening. Dorsal skin biopsies were taken every 4th week after Omniscan treatment and at sacrifice. Three skin samples from similar areas were taken from the back of each mouse. Samples were fixed in 10% formalin. After dehydration, all tissue samples were embedded in paraffin and sectioned for hematoxylin and eosin (H&E), Perl’s Prussian blue (skin, liver, and kidney), and immunohistochemical staining. All histology and immunohistochemistry studies were conducted in the University of Arkansas for Medical Sciences dermatopathology lab. A qualified dermatopathologist with extensive expertise in reading NSF (KMH) and pathologist (JM) performed the histopathology reading and analysis in a blinded fashion according to the best-practice guidelines of the Society of Toxicologic Pathology [13]. Dermal thickness was assessed by measuring the dermal area with a drawing tool (Analysis 3.2, Software Imaging Systems, Muenster, Germany). The dermis was measured in 3 places from the top of the granular cell layer to the dermal/subcutaneous junction. Skin sections were stained for iron deposition using a Perl’s Prussian blue kit (Polyscience, Warrington, PA) according to the manufacturer’s instructions. After deparaffinization, skin sections were incubated in equal amounts of 4% potassium ferrocyanide and 4% HCl solution for 20 minutes. After incubation, slides were washed twice with 1X PBS and counterstained with Nuclear Fast Red solution (Sigma-Aldrich, Saint Louis, MO) for 30 minutes. Images were captured using a Nikon Eclipse E800 microscope with a Cool SNAP camera (Nikon Metrology, Inc., Brighton, MI).

### Immunohistochemistry

Immunohistochemistry staining was performed on 5–6 μm dorsal skin sections. Antigen retrieval was performed by incubation (100°C, 20 minutes) with Antigen Unmasker Plus (ID Laboratories, London, Ontario, Canada) using a biotinylated secondary antibody/extrAvidin-horseradish peroxidase approach. CD163, CD34, procollagen-1, ferroportin, and hepcidin were detected by using appropriate antibodies (Details of the antibodies are given in Table 1). A goat anti-mouse, goat anti-rabbit, secondary antibody linked to horseradish peroxidase (Dako North America, Inc., Carpenteria, CA) was used with a peroxidase substrate kit (AEC, Vector Laboratories, Burlingame, CA). Slides were counterstained with haemotoxyline. For quantitative analysis of immunohistochemical staining, skin sections from 4 different mice from each group were used. Three random images of the epidermal field were taken from each section using a digital camera (Nikon Digital Sight DS-R-i1, Nikon Corporation, Japan) attached to a NIKON Eclipse 800 microscope at 440x magnification. The number of positively-stained cells /μm^2^ was determined using NIS-Elements Analysis software (Nikon). Quantitative analysis was performed by a trained pathologist (JM) blinded for the study.

**Table 1:** Primary antibodies used in this study. IHC—immunohistochemistry, WB—western blot.

Antibody	Species	Dilution used	Supplier	Catalog number
Anti-CD163	Rabbit (polyclonal)	1:100 IHC	Santa Cruz Biotechnology, Santa Cruz, CA	SC-33560, Clone M96
Anti-CD163	Mouse (monoclonal)	1:1,000 WB	Novus Biologicals, Littleton, CO	NPB2-36494
Anti-CD34	Mouse (monoclonal)	1:100 IHC	Santa Cruz Biotechnology	SC-74499
Anti-procollagen-1	Mouse (monoclonal)	1:100 IHC	Santa Cruz Biotechnology	Sc-25973
Anti-procollagen-1	Sheep (monoclonal)	1:1,000 WB	R&D Systems, Minneapolis, MN	AF6220
Anti-ferroportin/SLC40A1	Rabbit (polyclonal)	1:200 IHC	Abcam, Cambridge, MA	ab85370
Anti-ferroportin/SLC40A1	Rabbit (polyclonal)	1:1,000 WB	Novus Biologicals	NBP1-21502
Anti-hepcidin	Rabbit (polyclonal)	1:200 IHC	Abcam	ab81010
Anti-hepcidin	Goat (polyclonal)	1:1,000 WB	Novus Biologicals	NBP1-59337
GAPDH (HRP conjugated)	Rabbit (monoclonal)	1:2,000 WB	Cell Signaling Technology, Boston, MA	8884, Clone D16H11

### Biochemical analysis

Blood samples were collected for determination of hematological and biochemical parameters on day 0 (the first day of Omniscan treatment) and at sacrifice. Hematological parameters, specifically hemoglobin and hematocrit, were determined with a HEMAVET 950 FS (Drew Scientific, Inc., Waterbury, CT) in the VAMU. Blood was collected biweekly by retro-orbital approach under isoflurane anesthesia for plasma creatinine and BUN measurements (VetScan, Abaxis Veterinary Diagnostics, Union City, CA) in the VAMU during the study. Measurement of serum ferritin concentration was carried out by a mouse-specific, enzyme-linked immunosorbent assay (ELISA) by a commercially available kit (GenWay Biotech Inc., San Diego, CA) as instructed by the manufacturer.

### Cell culture and treatments

Commercially available human PBMC (AllCells, Emeryville, CA) were used for in vitro studies and were cultured as described previously [6]. Cells were cultured in complete growth medium containing Dulbecco’s Modified Eagle’s Medium (DMEM) (American Type Culture Collection, Manassas, VA), 10% heat-inactivated fetal bovine serum (American Type Culture Collection), 1% penicillin, streptomycin, and 1% L-glutamine (Invitrogen Life Technologies, Carlsbad, CA). PBMC were treated with 0.5 mM Omniscan for 8 days, as we had observed earlier Three injections of Omniscan (0.5 mmol/kg, tail IV, 100 μl volume) per week on alternate days were given to two groups of mice (one received deferiprone and the other received Omniscan only) that the effect of Omniscan on cell differentiation peaked at 8 days at a 0.5 mM dose. For iron chelation studies, cells were pretreated with various doses (from 25–150 μM) of deferiprone for 24 h before adding Omniscan to the culture.

In separate experiments, we determined the cellular toxicity induced by different doses of deferiprone alone, and cells treated with 0.5mM Omniscan and deferiprone. Cell culture media from control and treated cultures were analyzed for the percentage of lactate dehydrogenase (LDH) released using a commercially available colorimetric kit, the Cytoscan Lactate Dehydrogenase Cytotoxicity Assay kit from G-Biosciences (St. Louis, MO), according to the manufacturer's instructions. In brief, total PBMC were seeded in 96-well microplates at 20 x 10^3^ cells/well in DMEM containing 10% fetal bovine serum. After 8 days of incubation, adherent cells were lysed and the cell culture supernatants analyzed for lactate dehydrogenase concentration.

### Bleomycin-detectable iron assay

At the end of the experiments, cell culture medium was collected and catalytic iron (capable of catalyzing free-radical reactions) was measured by the bleomycin assay, as described earlier [13]. Catalytic iron released into the culture medium was measured as follows: One milliliter of the reaction mixture contained in order: 0.5 ml of calf thymus DNA (1 mg/ml), 0.05 or 0.1 ml of bleomycin sulfate (1 mg/ml), 0.1 ml of MgCl2 (50 mM), 0.1 ml of sample, 0.1 ml of Chelex-treated pyrogen-free water, 0.1 ml of ascorbic acid (8 mM), and either 0.05 ml of HCl (10 mM) or 0.1 ml of imidazole (1.0 M, pH 7.3) to adjust to pH 7.4. Sample blanks were identical except that bleomycin was omitted. The samples were then incubated at 37°C for two hours with shaking. The reaction was stopped by adding 0.1 M EDTA mixed with 1 ml of thiobarbituric acid (1% wt/vol in 50 mM NaOH) and 1 ml of 25% HCl (vol/vol). The reaction mixture was then heated at 100°C for 15 minutes, cooled, and the resulting chromogen measured using a spectrophotometer by its absorbance at 532 nm. A standard curve was prepared using known amounts of FeCl3 in Chelex-treated pyrogen-free water. The amount of bleomycin-detectable iron in the test sample was calculated from the standard curve and the results expressed as nmol/mg cellular protein recovered from the cell monolayer. Protein was measured by the use of a Bio-Rad Laboratories, Inc. (Hercules, CA) reagent. All reagents except for the sample to test were prepared in Chelex-treated pyrogen-free water and shaken with Chelex-100 to remove as much contaminating iron as possible.

### Immunofluorescence study

Total PBMC were seeded in fibronectin-coated 4-well BD Biocoat chamber slides (Fisher Scientific) at a density of 150 x 103 cells/chamber in complete growth medium and treated with 0.5 mM gadolinium for 8 days. Immunofluorescence studies were conducted to check the expression of CD163, CD34 and procollagen-, prolyl 4-hydroxylase (All from Santa Cruz Biotechnology, Inc., Santa Cruz, CA), and ferroportin and hepcidin (Abcam, Cambridge, MA). After treatment, cells were washed with 1xPBS and fixed with 4% neutral buffer formaldehyde for 15 min at room temperature. Slides were washed with 1xPBS three times for 5 min each and rinsed with 100% alcohol. Nonspecific sites were blocked by incubating the cells in 0.1% triton x-100 and 5% goat serum for 30 min at room temperature and washed 3 times with 1xPBS for 5 min each. Cells were incubated with 2–10 μg/ml primary antibodies, (described above) diluted in 1% BSA and 1xPBS at 37°C for 1h. Cells were washed 3 times with 1xPBS for 5 min each. 2–10 μg/ml secondary antibodies, either FITC (fluorescein isothiocyanate) or TR (Texas Red) conjugated (Santa Cruz Biotechnology, Inc.) and/or Alexa Fluor 488 and 546 anti-goat/rabbit and mouse IgG (Molecular Probes, Life Technologies, Grand Island NY), diluted with 5% goat serum in 1xPBS and added to the cells and incubated at 37°C for 1 h. For negative controls, cells were incubated with IgG only, without specific primary antibodies. Cells were washed 5 times with 1xPBS for 5 min each and mounted with Vectashield mounting media (Vector Laboratories Inc. Burlingame, CA) containing DAPI for counterstaining the nuclei. Slides were observed under a fluorescence microscope (Olympus America, Melville, NY).

### Western blot analysis

SDS-PAGE for CD163, CD34, ferroportin and procollagen-1 were carried out in 4–12% Bis-Tris NuPage separating gel (Invitrogen Life Technologies; Carlsbad, CA). For hepcidin, protein samples were separated on 10–20% Nu-Page Bis-tris gel run with NuPAGE MES SDS running buffer (Life Technologies). Equal amounts of protein (30 μg) were loaded into each lane, and the fractionated protein was electroblotted onto nitrocellulose membranes at 30 V for 1 h at room temperature. For CD163 and CD34, membranes were blocked in 5% clear milk in 1X TBS with 0.01% tween 20 (Pierce, Rockford, IL), and for ferroportin, hepcidin, and procollagen-1, 5% clear milk+2% BSA 1X TBS with 0.01% tween 20 for 1 h at room temperature and then probed with 1:1000 primary antibodies (details of the primary antibodies are listed in Table 1) diluted to in blocking solutions and incubated for overnight at 4°C using gentle shaking. Membranes were washed five times (5 min each) with Tris-buffered saline-Tween 20 [TBST; 20 mM Tris HCl (pH 7.6), 137 mM NaCl, and 0.2% (vol/vol) Tween 20] and incubated with horseradish peroxidase-coupled anti-IgG (anti-sheep-from R&D Systems, anti-mouse and anti-goat- from Novus Biologicals, and Santa Cruz Biotechnology (anti-mouse), and anti-rabbit from Abcam (secondary antibody, dilution 1:2000) for 1 h at room temperature and then washed three times. Enhanced Chemiluminescence (SuperSignal West Pico Chemiluminescent Substrate, Thermo Fisher Scientific, IL, USA) and fluorescence detection steps were followed per the manufacturer’s instructions for visualization of the bands. Bands were quantified using an Alpha Innotech ChemiImager 5500 (Alpha Inotech Corporation, CA). For the loading control, at the end of the experiments, nitrocellulose membranes were stripped with restore western blot stripping buffer (Thermo Fisher Scientific) and re-probed with HRP conjugated anti-GAPDH antibody (1:1000 dilution, Cell Signaling Technology).

### Statistical analysis

Statistical analysis was conducted using Prism 6.0 for Windows (GraphPad, SanDiego, CA). Data for all outcomes were summarized by group as the mean ±SD (standard deviation). For each outcome, the differences between group means were assessed for statistical significance at alpha = 0.05 via one-way ANOVA with the Tukey-Kramer post-hoc procedure to control for the multiple comparisons. Data were considered significant at P < 0.05.

## Results

### Omniscan-induced NSF in a murine CKD model

The values of BUN and creatinine in the control and sham groups, and in the CKD groups before injection of Omniscan and at sacrifice, are shown in Table 2. Serum creatinine and BUN were significantly higher (p <0.001) than in sham mice at the time of sacrifice (Table 2). During the study, dorsal skin biopsies were taken every 4^th^ week (as established in our preliminary study). The dermatopathology lab analyzed the samples histopathologically and by immunohistochemistry (KMH). Mice treated with 0.5 mmol/kg started developing changes in skin from the 10th week (presence of CD34^+^ and procollagen^+^ and fibroblast-like cells). By the 16th week more clinically visible changes of NSF were evident including hair loss, indurations, and skin tightening. Skin biopsy samples showed a significant increase in CD34, CD163^+^ dermal spindle cells, and thick collagen bundles with skin thickness, as determined by immunohistochemical and histopathological analysis.

**Table 2:** 

	Normal	SHAM	CKD	CKD + Omni	CKD +Omni + DFP
	(n = 4)	(n = 5)	(n = 4)	(n = 8)	(n = 9)
Body weight (g)	28±0.3	27.8±0.9	24.4±-0.8	22.7±1.3*	24.5±1.4
Hb (g/dl)	15±0.8	12.3±2.3	10.9±1.3	12.0±0.8	11.5±0.83
HCT (%)	55±3	48±8	44±6	48±4	46±4
WBC (k/μl)	9.2±3	7.9±2.2	9.0±6.4	13.9±6.0	9.1±4.4
Platelets (k/μl)	560±78	405±251	206±127	334±208	186±87
BUN (mg/dl) one week after nephrectomy at sacrifice	(16±9) 17±2	(16±2)17±1	(78±12)***55±6**	(77±8)***55±7**	(78±9)***46±5**
Creatinine (mg/dl) one week after nephrectomy at sacrifice	(0.13±0.09)0.2±0.02	(0.19±0.2)0.2±0.03	**(**0.49±0.1)**0.4±-0.04*	**(**0.48±0.1)**0.35±0.2*	**(**0.48±0.1)**0.3±0.08*
Ferritin (ng/ml)	17±1.8	35±9.5	26±6.6	31±11.0	19±6.0†

*p<0.05

**p<0.001, and

*** p<0.0001 as compared to sham mice.

† p<0.05 as compared to CKD plus Omniscan.

The data presented here are from the results obtained at the time of sacrifice. Values are means± SD, *** *p*<0.0001 **p<0.001, *p<0.05 as compared to sham mice by unpaired two-tailed Student’s t-test. †*p*<0.05 for ferritin as compared to Omniscan-treated mice with CKD. (n = 5 sham, n = 4 CKD control, n = 8 mice with CKD for Omniscan-treated group, and n = 9 for mice with CKD for Omniscan plus DFP-treated group).

**Fig 2:**
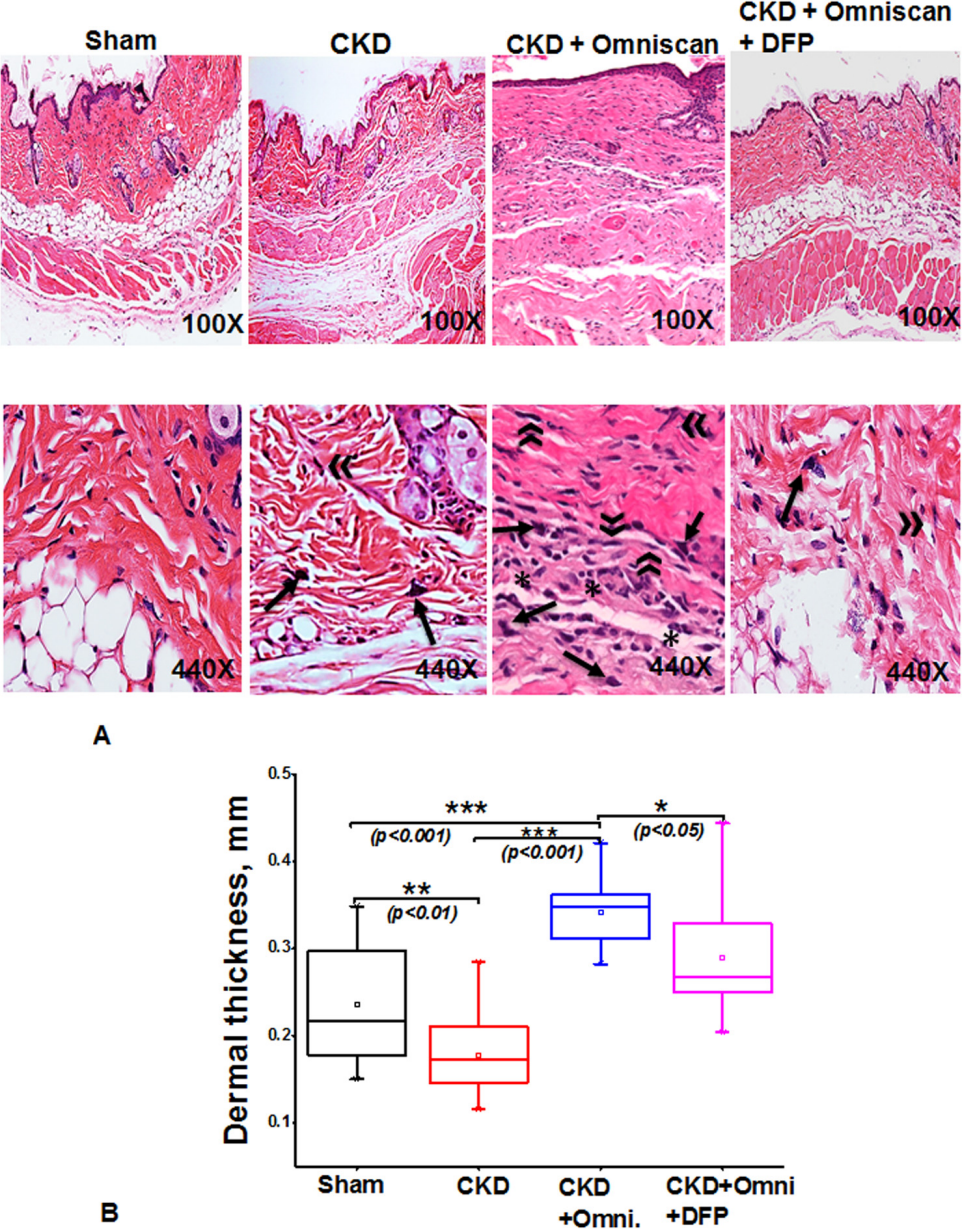
Omniscan-induced NSF in a Balb/C mouse model. **(A)** Histopathological findings in the skin of mice with CKD treated with either Omniscan only or Omniscan with deferiprone. Representative images are of dorsal skin biopsies of each group at lower (100X) and higher (440X) magnification H&E stained sections show Omniscan induces dermal thickness, lipoatrophy, inflammatory cells, dermal fibrosis, and a marked increase in cell number and density of collagen bundles (signs of NSF), and deferiprone visibly prevents NSF in mice skin. For fibrocyte-like cells, an arrow represents dendritic cells and an asterisk shows inflammatory cells. (**B)** A box-whisker chart shows data of the dermal thickness for the sham, CKD, CKD with Omniscan, and CKD with Omniscan and deferiprone (n = 4 for each group). *p<0.05, **p<0.01, ***p<0.001, as compared to indicated group. Box: 2–75 percentile; whiskers: 5–95 percentile; horizontal line: median, square: mean. The significance of differences among groups was evaluated using a one-way analysis of variance (ANOVA) with a posthoc Tukey-Kramer test.

**Fig 3:**
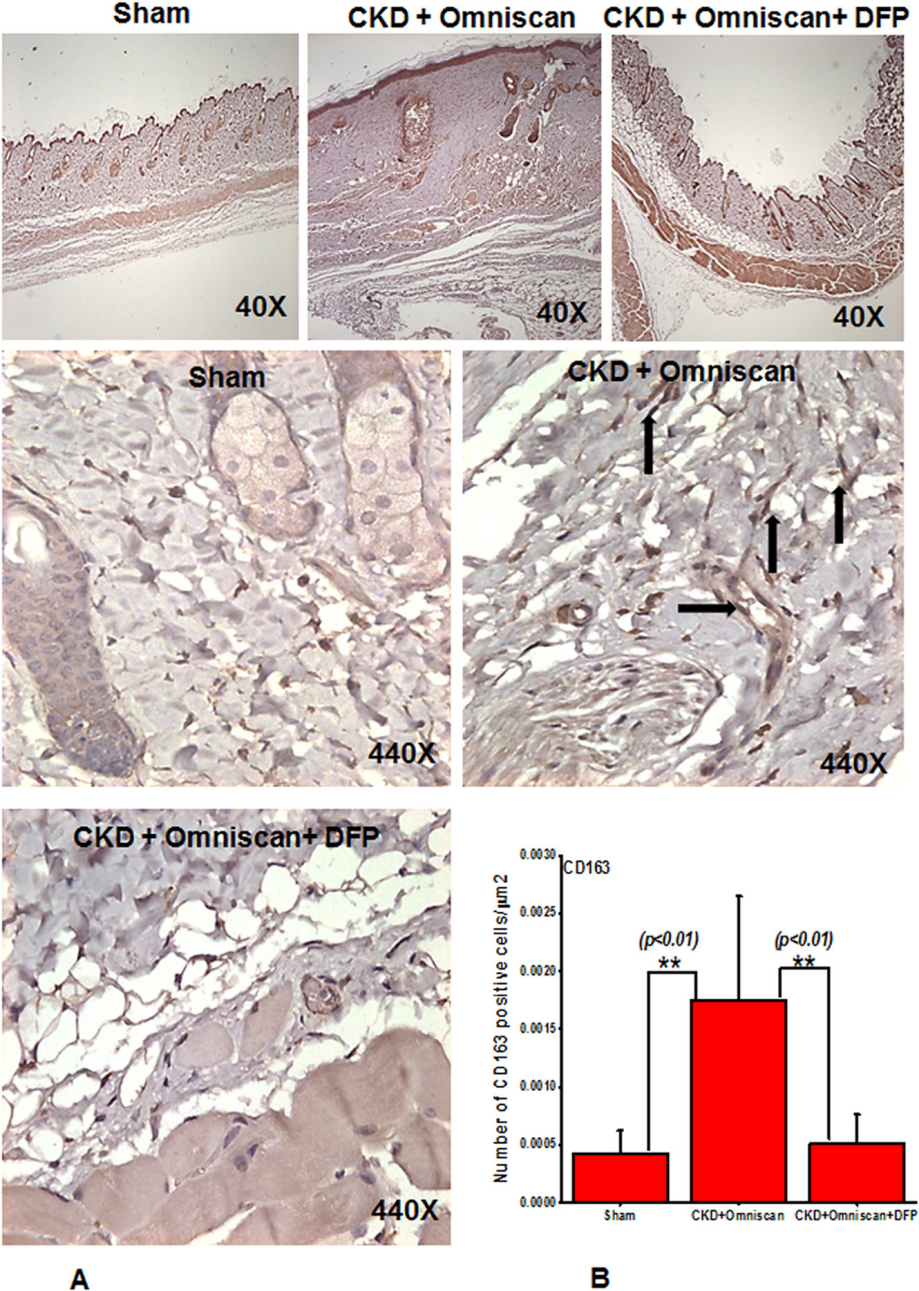
Deferiprone down-regulates Omniscan-induced CD163^+^ cells in NSF skin as shown by immunohistochemistry (Panel A). Immunohistochemistry staining shows deferiprone treatment decreases Omniscan-induced CD163^+^. The top panels (40X) show the comparative skin thickness from each group. Panel B demonstrates the quantitative analysis of the number of positively stained cells in μm^2^. (**p<0.01 Omniscan alone compared with control or Omniscan treated compared with deferiprone and Omniscan).

**Fig 4:**
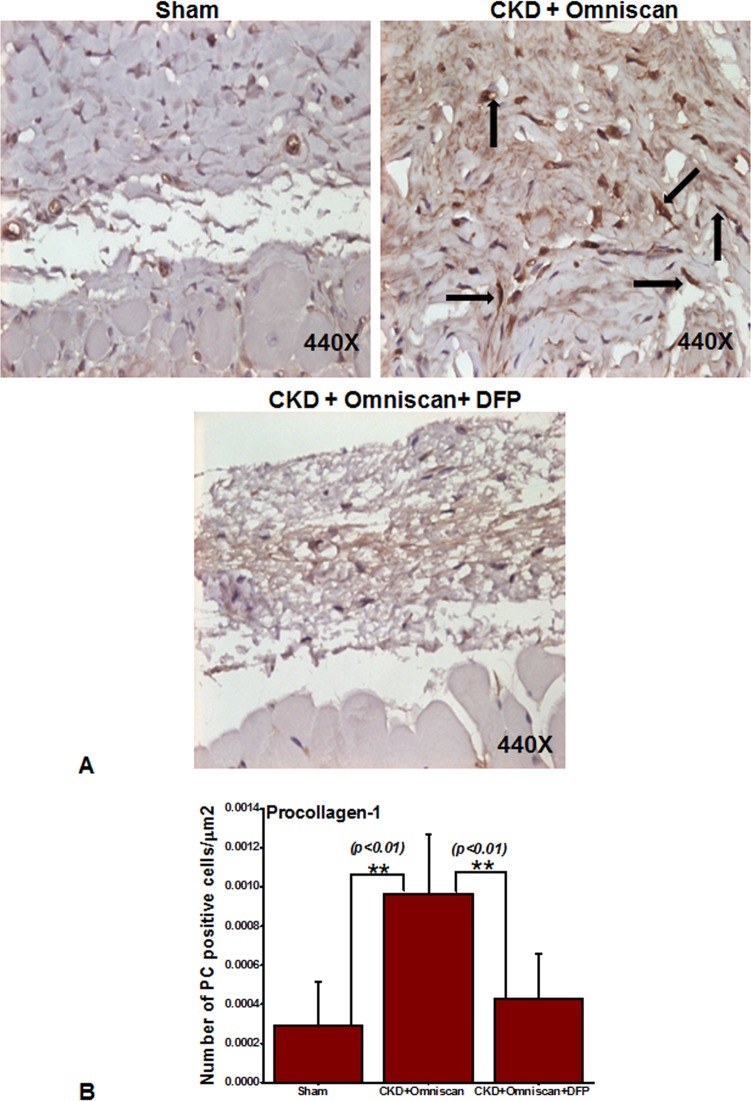
Deferiprone down-regulates Omniscan-induced procollagen-1^+^ cells in NSF skin as shown by immunohistochemistry. As indicated by arrows, Omniscan induced procollagen-1^+^ cells and accumulation of collagen bundles in the skin of CKD mice. Quantitative analysis of the number of positively stained cells is in μm^2^. (**p<0.01 Omniscan alone compared with control or Omniscan-treated compared with deferiprone and Omniscan).

**Fig 5:**
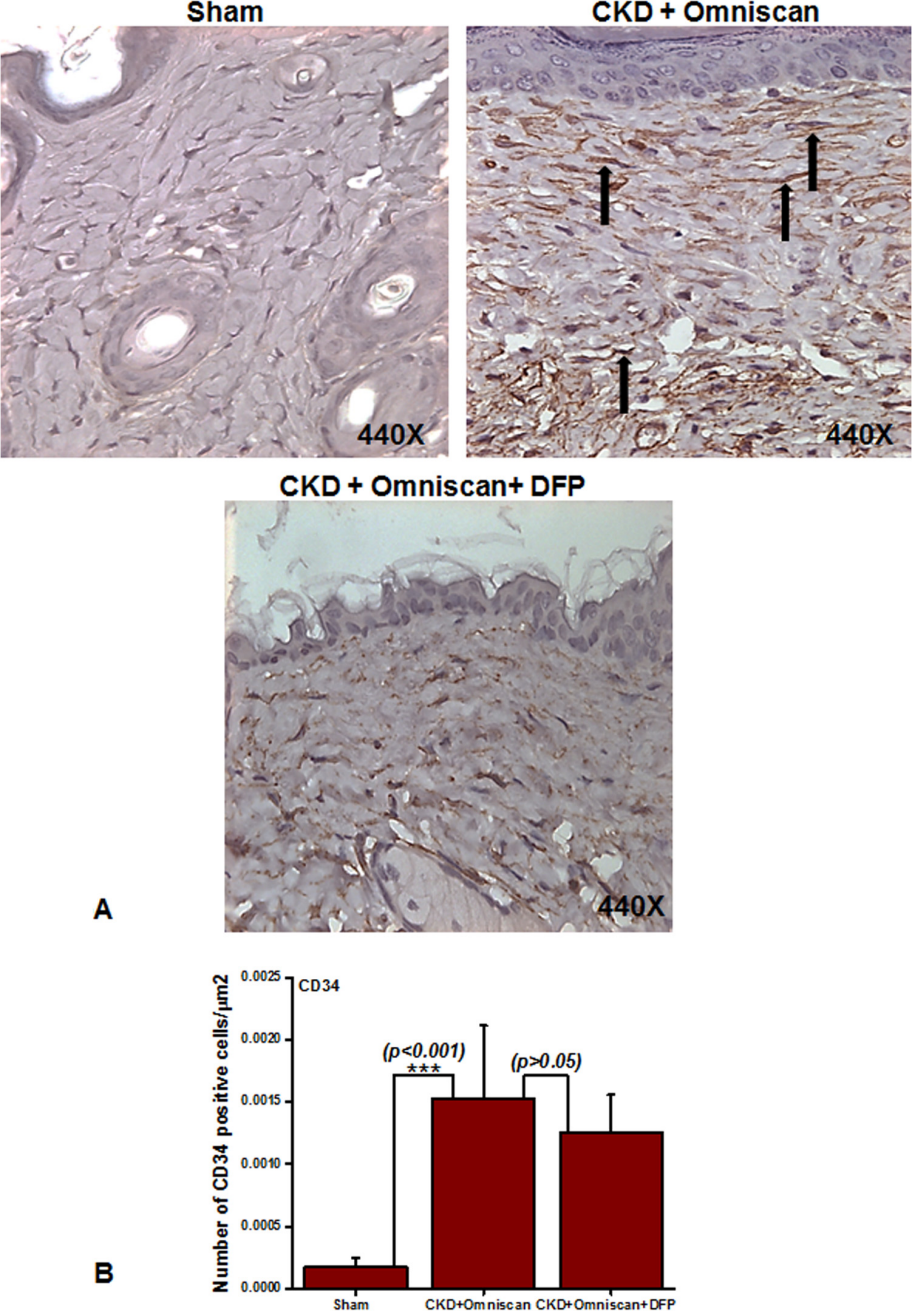
Expression of CD34+ cells in NSF skin as shown by immunohistochemistry. Panel A shows (as indicated by arrows) more CD34^+^ cells in CKD skin of Omniscan-injected mice. This was significant statistically (***p<0.001, Panel B). Quantitative analysis shows that CD34 was not reduced significantly with deferiprone treatment (Panel B).

Histopathological sections of the Omniscan-treated group’s (Group 4) skin demonstrated dermal fibrosis with increased skin thickness, cellularity (increase in spindle-shaped cells, dendritic-like cells, Fig 2A). Quantitative analysis of the skin thickness showed a very significant (*p*<0.001) difference between mice with CKD and CKD mice injected with Omniscan (Fig 2B). As shown in Figs 3–5, skin sections of Omniscan-treated mice show markedly up-regulated expression for fibrocyte markers including CD163, procollagen-1, and CD34, characteristics of NSF. Quantitative analysis showed a significant increase in CD163 and PC expression in the Omniscan treated group (*p*<0.01).

### Iron chelator therapy prevents Omniscan-induced NSF

The body weights of the Omniscan-treated CKD mice were significantly less (*p* < 0.01) than the control and DFP-treated CKD mice (Table 2). There were no significant differences in hemoglobin and hematocrit levels between the CKD mice, the CKD mice treated with Omniscan, and the CKD mice treated with Omniscan and deferiprone. Serum ferritin levels were significantly less (*p* < 0.05) in the deferiprone-treated mice (19 ng/ml) compared to both the CKD (26 ng/ml) and CKD plus Omniscan groups (32 ng/ml).

Deferiprone provided significant macroscopic protection against Omniscan-induced NSF including reduction in redness, loss of hair, ulceration, and skin tightening. A histopathological study confirmed the protective effects of deferiprone. In deferiprone-treated mice, subcutaneous adipose tissues were well preserved (Fig 2A). Quantitative measurements of skin biopsies showed a significant decrease (*p*<0.05) in thickness in Omniscan plus deferiprone-treated mice with CKD compared to the Omniscan-only treated group (Fig 2B). Markers CD163 and procollagen showed a marked reduction after deferiprone treatment as observed by immunohistochemistry staining (Figs 3A and 4A) and quantitative analysis (*p*<0.01) (Figs 3B and 4B). CD34 was not reduced significantly with deferiprone treatment (Fig 5B). The water consumption for each group was similar.

**Fig 6:**
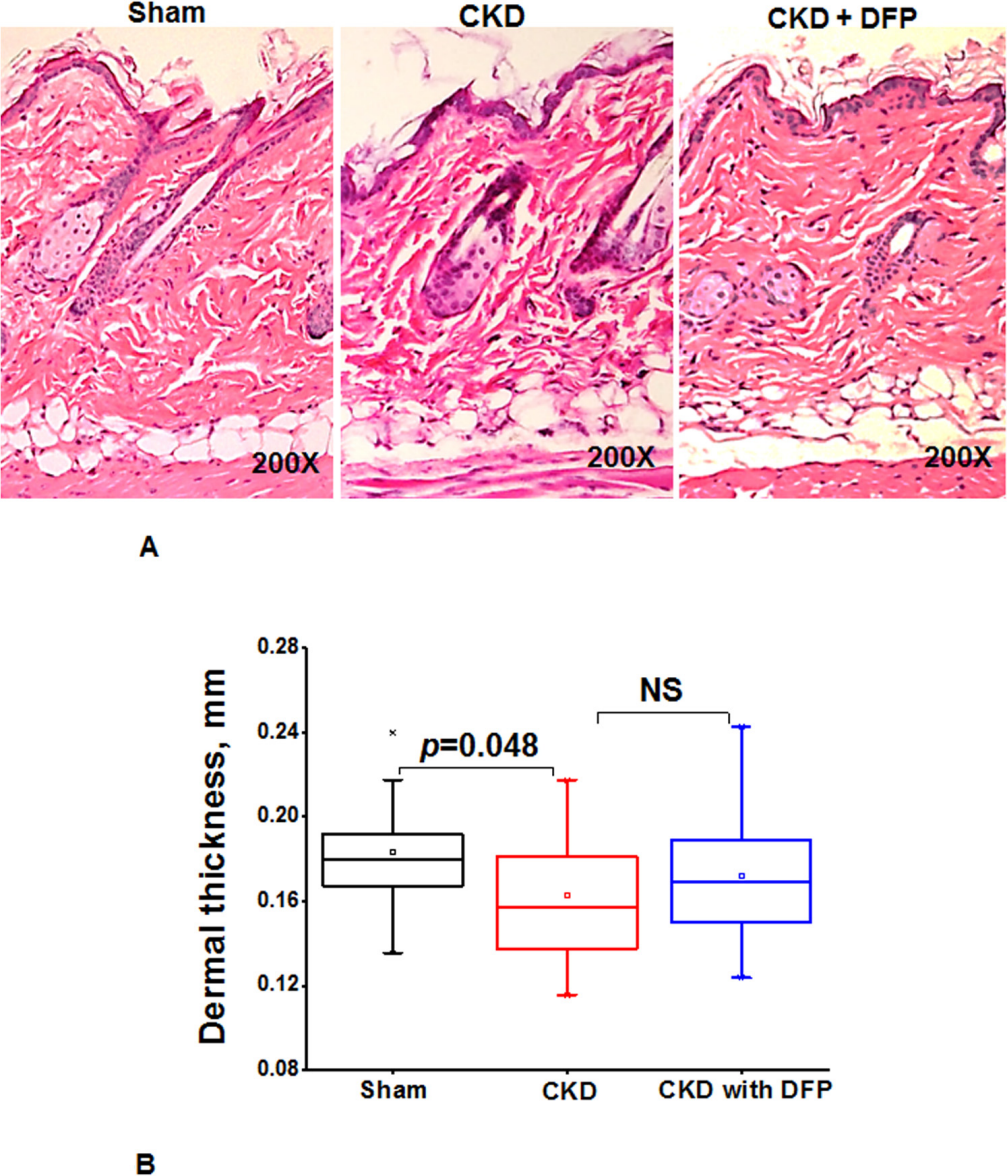
Deferiprone inhibits histopathological signs in skin of CKDmice. **(A)** Histopathological findings in the skin of mice with CKD and CKD mice treated with 125 mg/kg deferiprone for 16 weeks. Representative images are of dorsal skin biopsies of each group at 200X magnification H&E stained sections. **(B)** A box-whisker chart shows the data of the dermal thickness for the sham, CKD, CKD mice with deferiprone (n = 4 for sham, n = 10 for CKD, n = 12 for CKD mice treated with deferiprone). Data shows there was no significant difference between CKD and mice treated with deferiprone, NS = not significant. Box: 25–75 percentile; whiskers: 5–95 percentile; horizontal line: median, square: mean. The significance of differences among groups was evaluated by a one-way analysis of variance (ANOVA) and with a posthoc Tukey-Kramer test.

In a separate experiment we previously examined the effect of deferiprone (125mg/kg) administered for 16 weeks on skin histology in CKD mice. CKD mice had reduced skin thickness compared to controls, and deferiprone had a minor effect on skin thickness (Fig 6).

### Effect of iron chelation on Omniscan-induced tissue iron accumulation and tissue ferroportin expression

**Fig 7:**
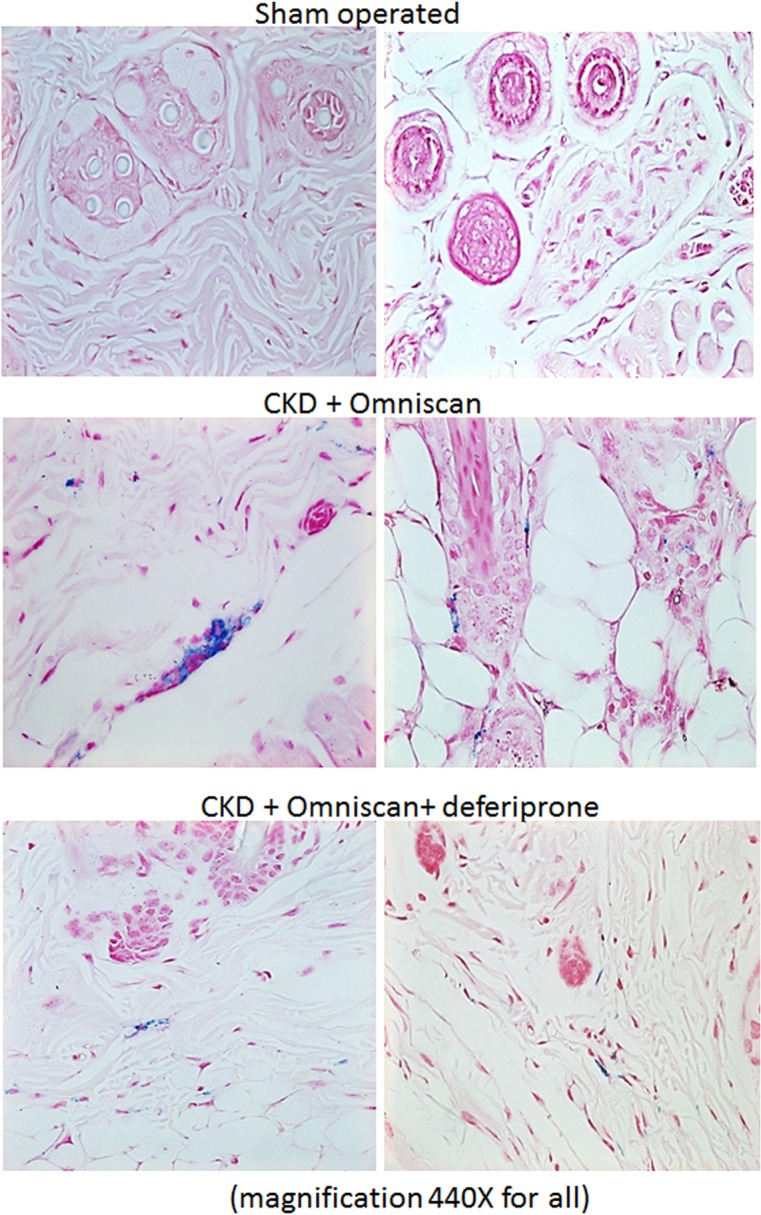
Presence of iron in NSF skin. A bright-field image shows Prussian Blue positive staining for presence of iron in mouse skin injected with 0.5 mM Omniscan and treated with 125 mg/kg deferiprone. Sections were counterstained with Nuclear Fast Red (magnification, 440X for all).

**Fig 8:**
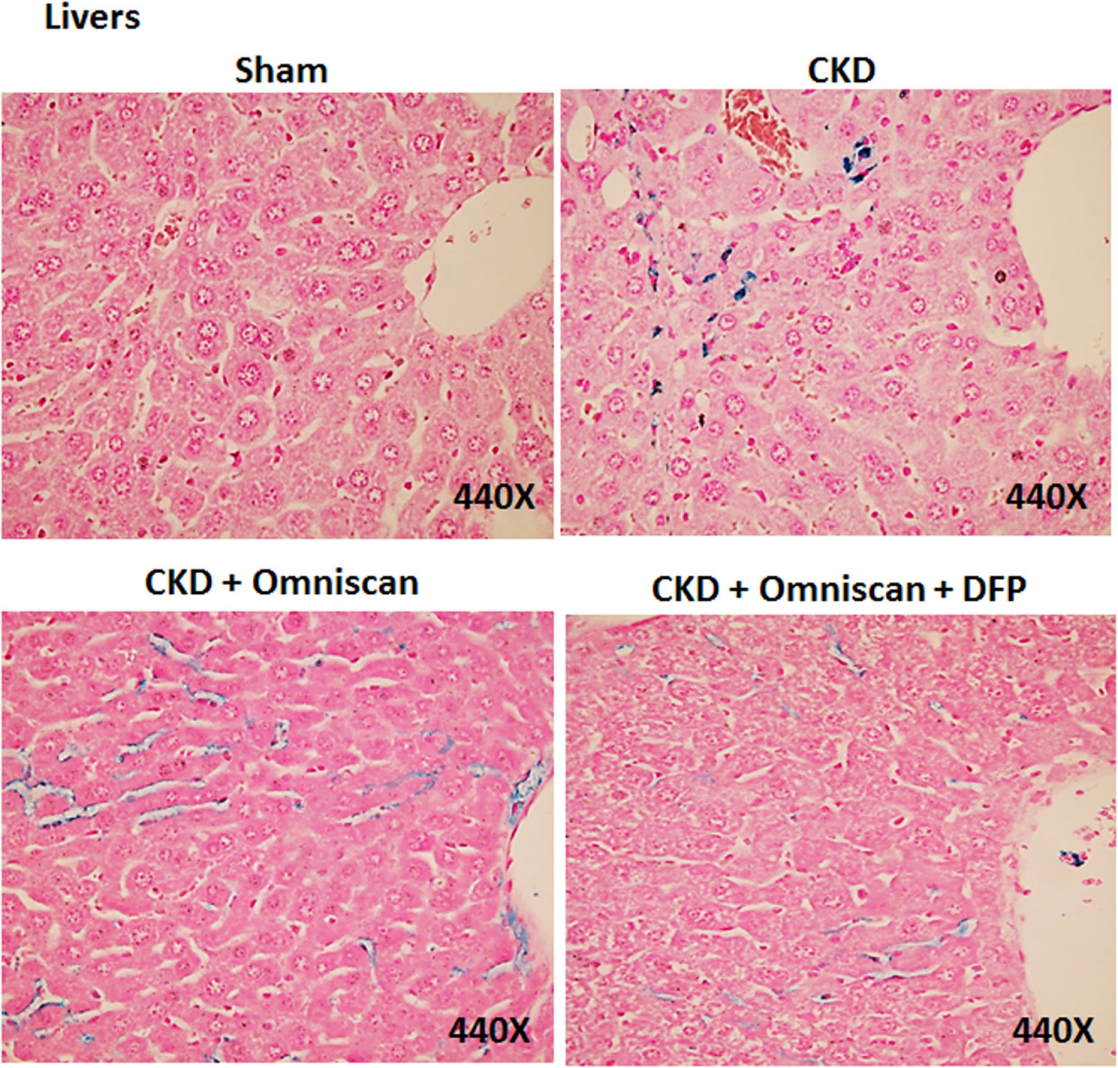
Iron deposition in liver. As shown, Omniscan-injected mice have more iron staining in liver sinusoidal Kupffer cells. Deferiprone treatment reduced the iron-positive cells markedly.

**Fig 9:**
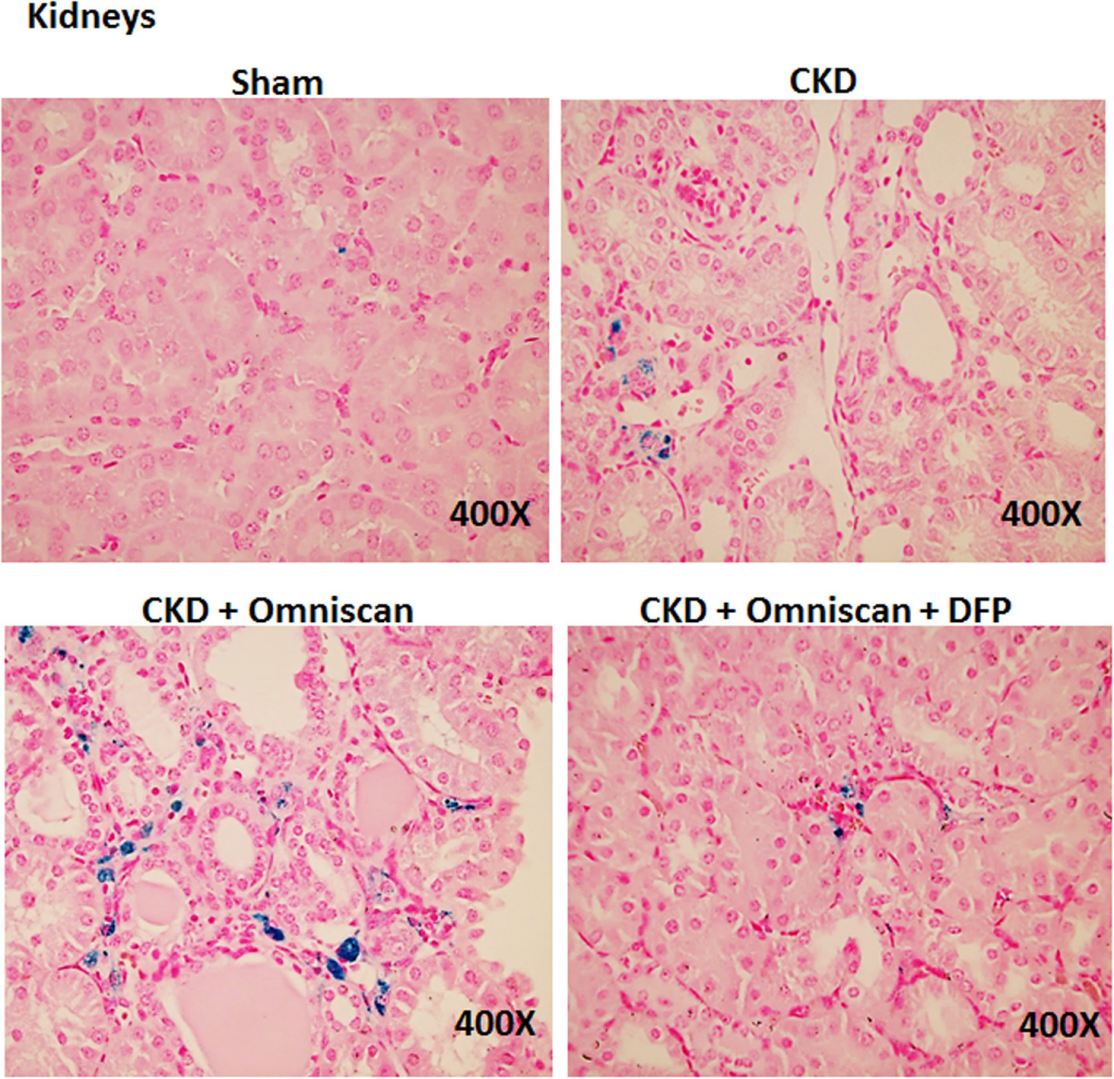
Iron deposition in kidneys. Omniscan-injected mice have more iron staining in the interstitial cells of kidneys, followed by the CKD-only group. Deferiprone treatment reduced the iron staining markedly in the kidneys of Omniscan-induced CKD mice.

Skin, liver, and kidney sections from Groups 2–5 were further stained with Perl’s blue to examine iron accumulation. In Omniscan-treated mice, positive blue granular staining was detected in individual as well as small groups of fibrocyte-like cells in the dermis (Fig 7). In the liver and kidney tissues, maximum iron accumulation was observed in Omniscan-injected mice. Iron accumulation is apparent in liver sinusoidal macrophages (Kupffer cells) in the section from CKD and CKD plus Omniscan-injected mice. When compared to the Omniscan group, in the deferiprone plus Omniscan-treated mice, iron staining was markedly reduced and iron deposition was minimal, with only occasional positive staining by any fibrocyte-like cells in the skin (Fig 7), liver (Fig 8), and kidneys (Fig 9). No detectable iron-positive cells or granules were evident in any sham-operated mice skin (Fig 7), liver, or kidney sections (Figs 8 and 9).

**Fig 10:**
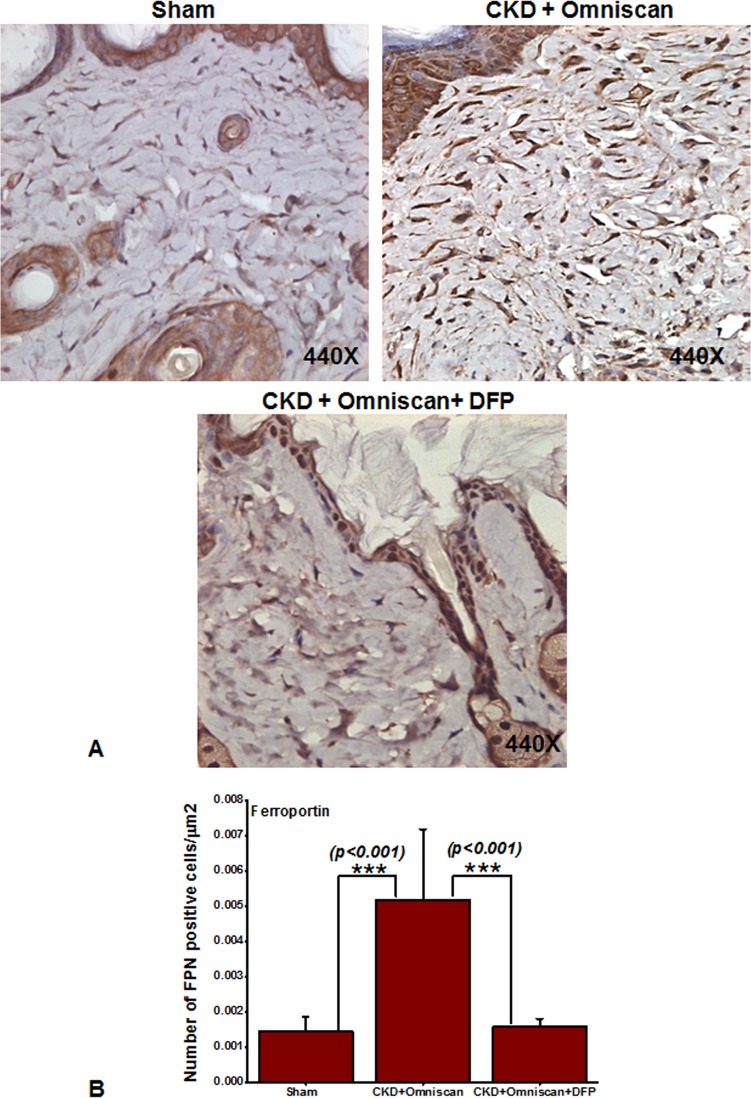
Ferroportin in Omniscan-injected mice. Omniscan significantly induces the iron transport protein “ferroportin” in mouse skin. Deferiprone treatment markedly decreases Omniscan-induced ferroportin expression in the mouse skin biopsies. Panel B demonstrates the quantitative measurements of positive cells in μm^2^. Significance of the data was determined by ANOVA, followed by paired-group comparisons. (***p <0.001 Omniscan-treated compared with deferiprone and Omniscan, or compared with Omniscan alone and control).

**Fig 11:**
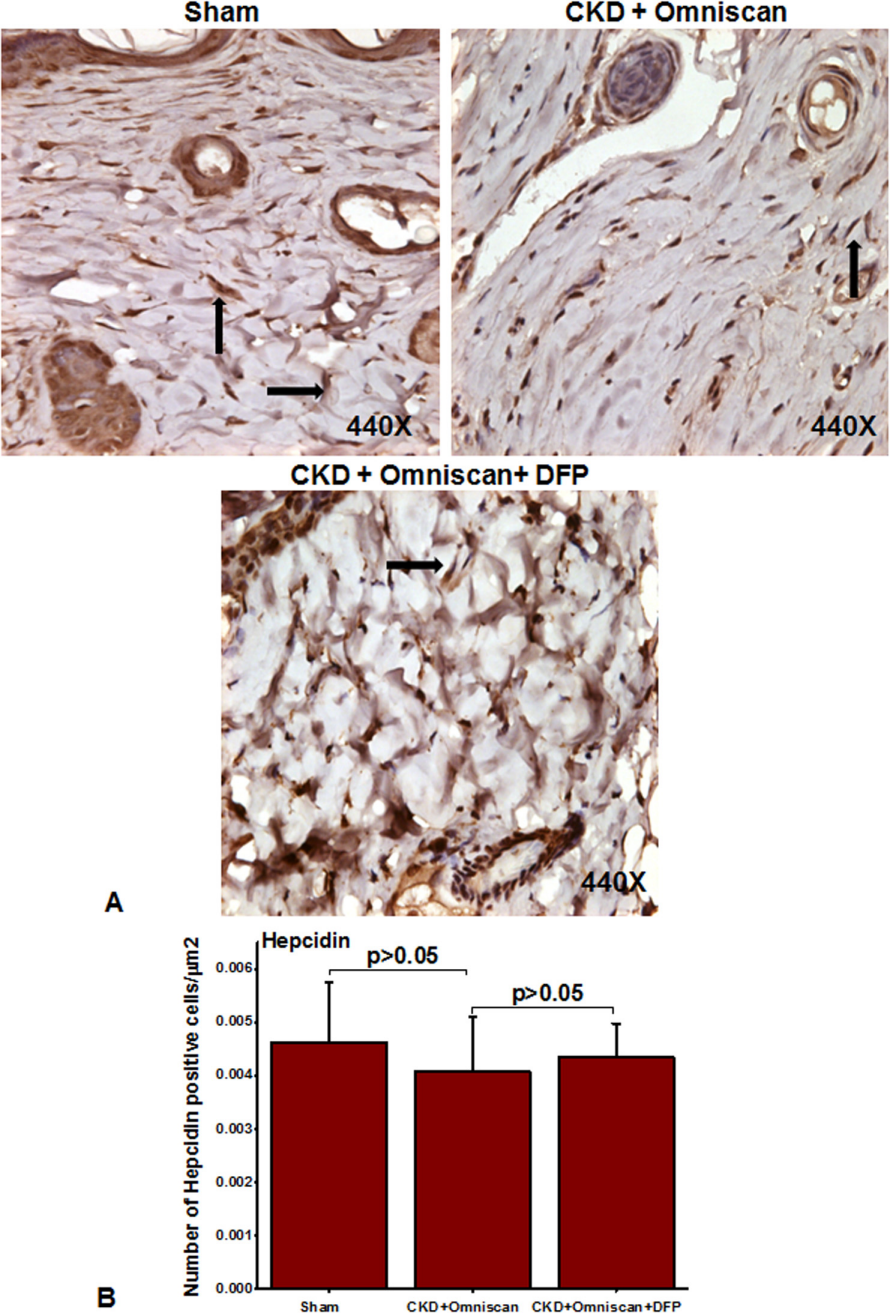
Expression of hepcidin in Omniscan-injected CKD mice. Hepcidin was slightly down-regulated after Omniscan treatment. Pretreatment with deferiprone partially increased hepcidin expression.

We further examined cellular localization and expression of ferroportin and hepcidin in the skin biopsies. As shown in Fig 10A, many fibrocyte-like spindle-shaped cells in the dermis of Omniscan-treated mice strongly expressed ferroportin, and hepcidin expression was lower than in sham-operated mice (Fig 11A). In mice that received deferiprone along with Omniscan, there was a marked reduction in the number of ferroportin-expressing fibrocyte-like cells accompanied by slight increased dermal hepcidin expression (Figs 10A and 11A). Quantitative analysis demonstrated that the number of cells positive for ferroportin were significantly (*p*<0.001) increased after Omniscan injections, whereas deferiprone treatment markedly (*p*<0.001) reduced ferroportin-expressing cells (Fig 10B). The number of hepcidin-expressing cells in sham and deferiprone-treated groups, and Omniscan-injected CKD mice, were not statistically significant (Fig 11B).

### Cell culture studies

We have recently shown that Omniscan induces the differentiation of human PBMC into ferroportin-expressing, fibrocyte-like, spindle-shaped cells [6]. In this study, we demonstrate that deferiprone treatment dramatically inhibited Omniscan-induced differentiation of PBMC into fibrocyte-like cells.

**Fig 12:**
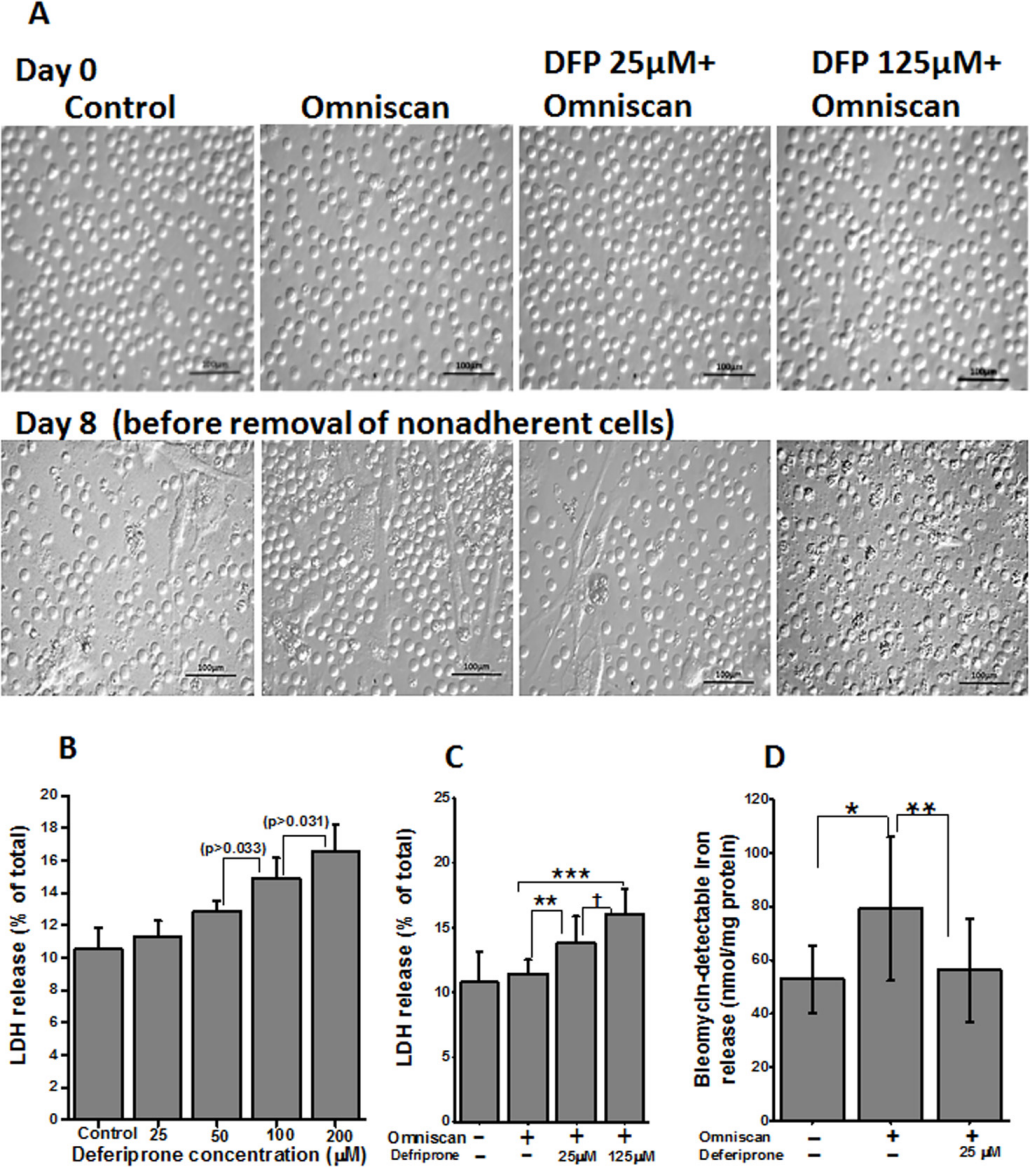
Effect of deferiprone on cell cytotoxicity. (**A)** Light microscopic images on day 0 shows an equal number of PBMCs for each group. (**B)** LDH release by PBMC after treatment with deferiprone for 8 days. Adherent cells were lysed and supernatant media were collected from these cultures and analyzed for LDH release. The data presented shows no significant cytotoxicity on lower concentrations of deferiprone from control. LDH release was slightly increased on 100 and 200 μM concentrations of deferiprone, *p* > 0.033 and *p* > 0.031, respectively. Data points represent means ± SD of 3 separate studies. (**C) LDH release by PBMC treated with 0.5 mmol of Omniscan and deferiprone together.** PBMC were treated with Omniscan and deferiprone for 8 days. LDH release was measured as above. As shown in the histogram, both doses of deferiprone used (25μM and 125μM) showed significant LDH release (**p<0.01, ***p<0.001, respectively) when compared to untreated cells, but, as seen in the lower panels of light microscopic images of the treated cells, 25 μM deferiprone with Omniscan was not toxic to the cells. Images were taken before removing nonadherent and dead cells. A higher dose showed toxicity (†*p*<0.01) and it was confirmed with a Trypan blue exclusion test. Data presented here are from 3 separate studies. (**D) Effect of deferiprone on Omniscan-induced catalytic iron release by PBMC.** Deferiprone treatment decreases the release of catalytic iron of Omniscan-treated human PBMC, as shown by use of a bleomycin-detectable iron assay. At the end of the experiments, the cell culture supernatant was collected for the measurement of bleomycin-detectable iron. Values are means ±SD, n = 3, *p <0.05 compared with control, **p <0.01, deferiprone and Omniscan treated compared with Omniscan alone.

In our present study we first evaluated the cytotoxic effect of deferiprone and deferiprone plus 0.5mM Omniscan on PBMC. As shown in Fig 12A, upper panels, an equal number of cells were plated on day 0. After treatment with different doses of deferiprone for 8 days, lactate dehydrogenase (LDH) release was checked for cytotoxicity. As shown in Fig 12B, no significant cytotoxicity was noted at lower doses of deferiprone used, compared to untreated control cells. The dose of Omniscan (0.5 mmol) was chosen based on our previous study in which we reported the cytotoxicity for Omniscan [6]. For this study, cells were treated with 0.5 mmol Omniscan and two doses of deferiprone. A lower dose (25 μm) of deferiprone resulted in a 13.8±2.0% increase in LDH release and a higher dose (125 μm) resulted in a 16±1.9% increase in LDH release, in comparison to control (10.7±2.3%) (Fig 12C). Statistical analysis showed the difference in LDH release between untreated cells and cells treated with Omniscan and deferiprone was significant (*p*<0.01) with both deferiprone doses used, but, as evident by the light microscopic images of the cells in culture after 8 days of treatment (Fig 12A, lower panels), the lower dose of deferiprone was not cytotoxic. The images were taken before removing the non-adherent cells. As previously reported, the total number of cells was increased in the Omniscan-treated plates. Treatment with 25 μM deferiprone prevented this increase and resulted in a similar number of cells that were Trypan blue-positive. The higher dose (125 μm) resulted in cytotoxicity (*p*<0.001) as compared to Omniscan alone, which is evident morphologically and confirmed with a Trypan blue exclusion test.

We examined the effect of Omniscan on catalytic iron. As shown in Fig 12D, Omniscan treatment significantly (p<0.05) increased catalytic iron release, while pre-treatment with 25 μM of deferiprone significantly (p<0.001) decreased catalytic iron release by the cells treated with Omniscan.

**Fig 13:**
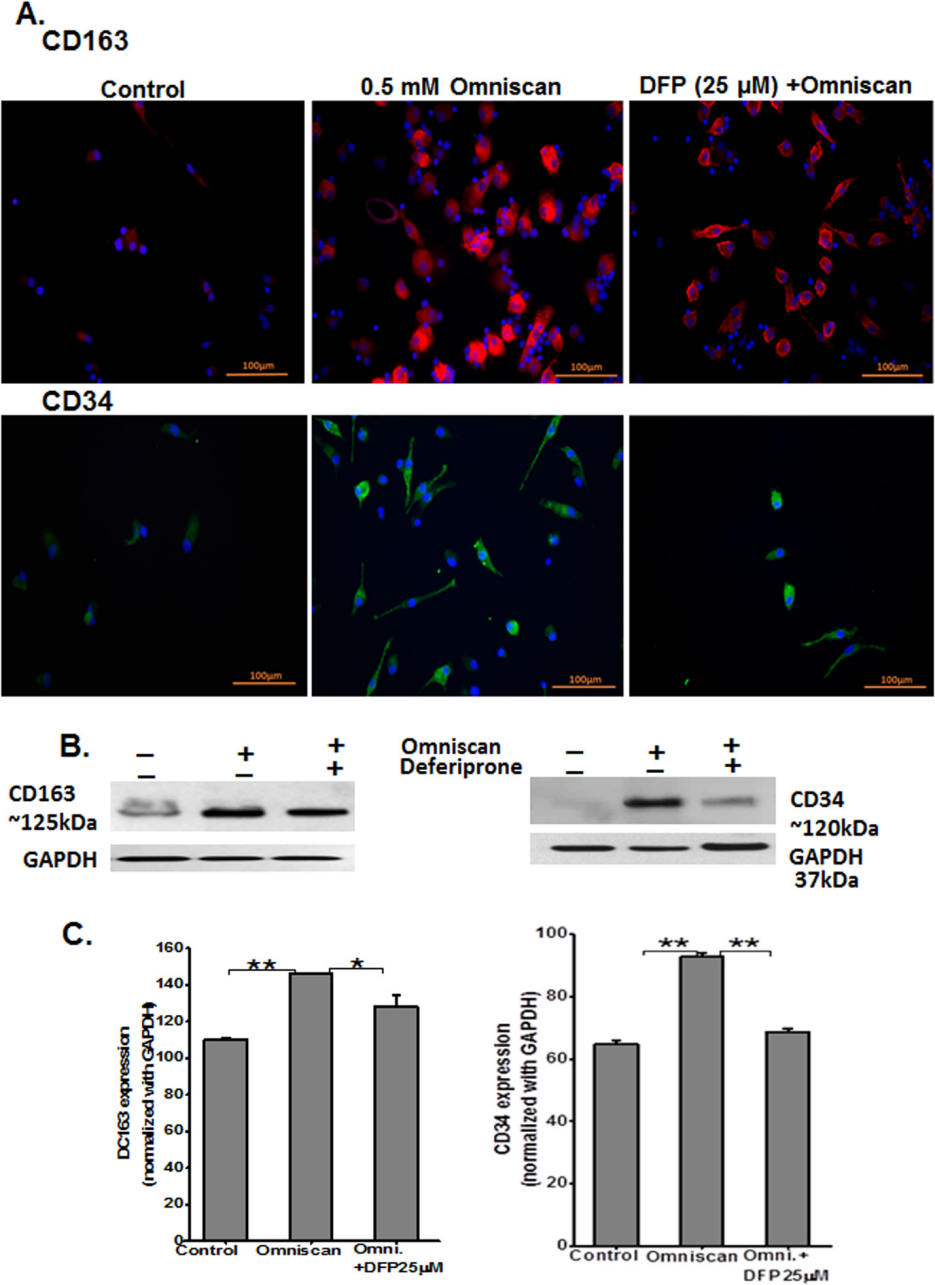
Expression of Omniscan-induced macrophage (CD163) and fibrotic markers (CD34) are iron-regulated, as shown by PBMC *in vitro*. Expression of CD163 and fibrotic markers are iron-regulated, as shown by immunofluorescence staining of human PBMC treated with 0.5 mM Omniscan plus deferiprone for 8 days. **(A)** Representative images show CD163 and CD34 expression by Omniscan, and 25 μM of deferiprone effectively inhibited differentiation of the cells and expression of these markers. (Scale bars 100 μm for all). Western blot and quantitative analysis for western blots are shown in Figs **(B)** and **(C).** GAPDH was used as loading control. Values are means ±SD, obtained from 3 separate experiments. Significance of the data was determined by ANOVA, followed by paired-group comparisons. (*p <0.05, for CD163, **p <0.01 for CD34 for Omniscan-treated compared with Omniscan plus deferiprone, **p <0.01, compared with Omniscan alone and control).

**Fig 14:**
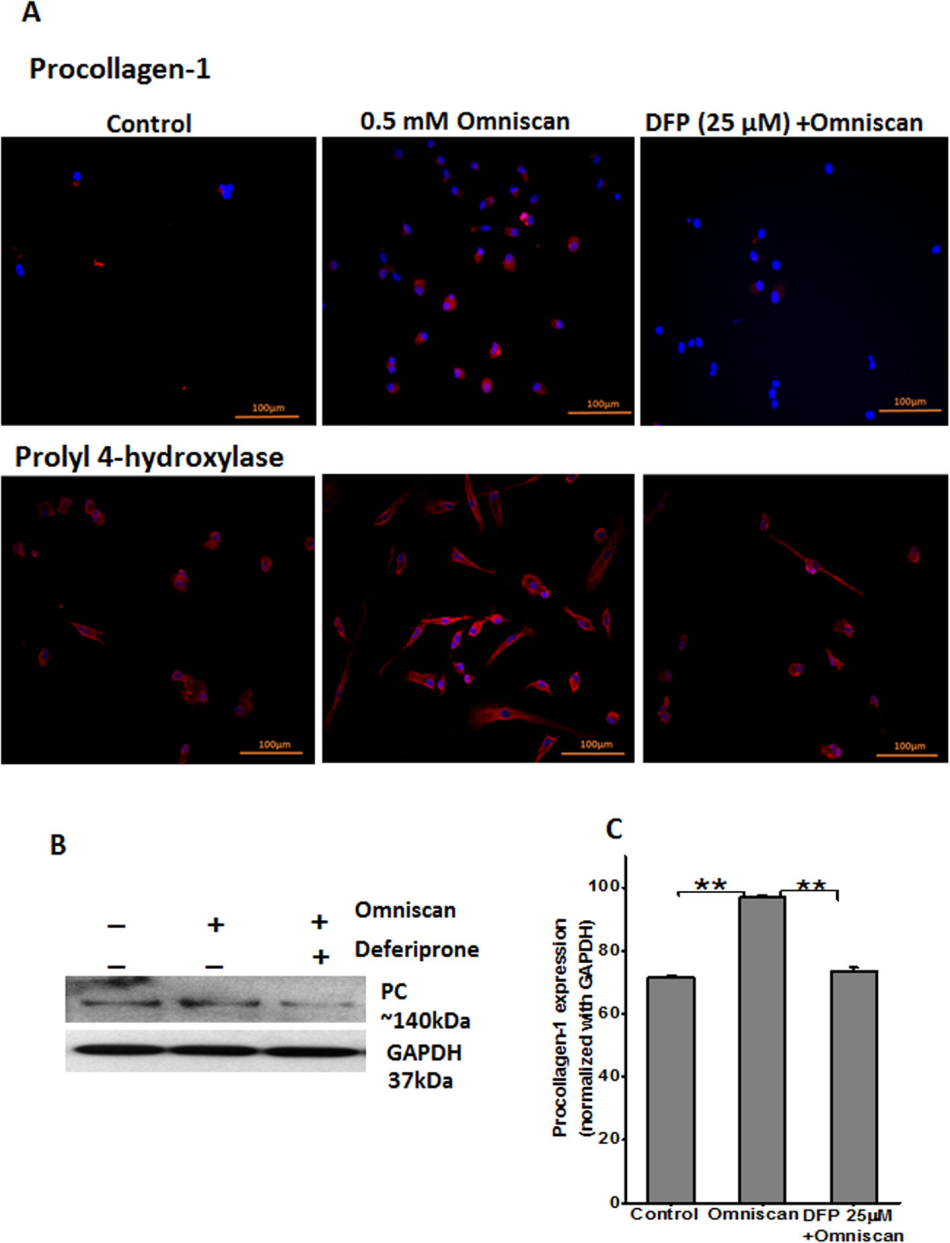
Expression of fibrotic markers procollagen-1 and prolylyl-4-hydroxylase as shown by immunofluorescence staining PBMC treated with Omniscan. **(A)** Representative images show procollagen-1 and prolylyl-4-hydroxylase expression by Omniscan and 25 μM of deferiprone effectively inhibited the expression and differentiation of the cells of these markers (scale bars 100 μm for all). Western blot and quantitative analysis for western blots are shown in Figs **(B)** and **(C).** Values are means ±SD, obtained from 3 separate experiments, Significance of the data was determined by ANOVA, followed by paired-group comparisons. (**p <0.01).

**Fig 15:**
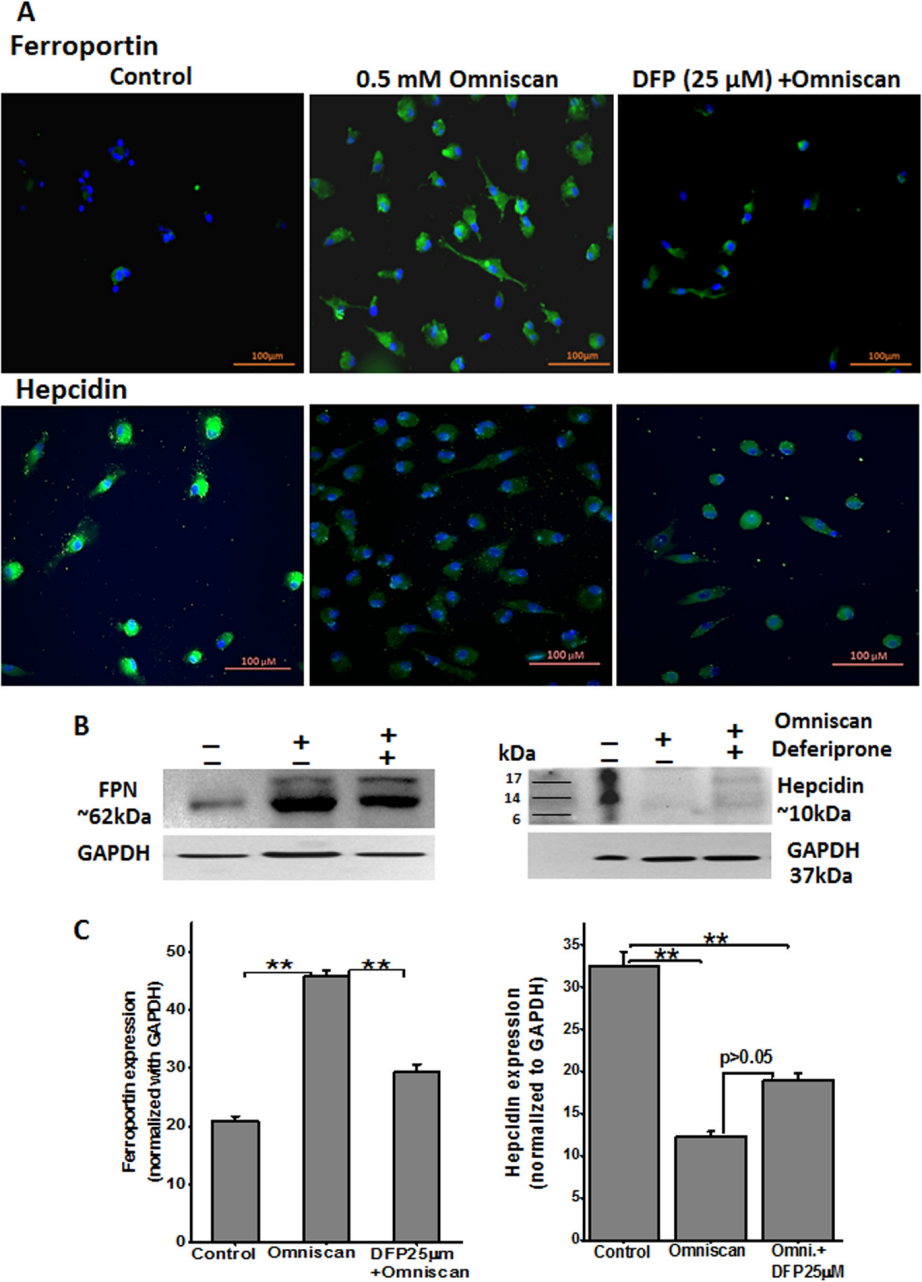
Omniscan-induced cells express iron metabolism proteins. **(A)** Expression of ferroportin and other iron regulatory protein hepcidin by human PBMC treated with 0.5 mM Omniscan and deferiprone for 8 days, as shown by immunocytochemistry staining. Representative images are shown. Deferiprone treatment significantly decreased Omniscan-induced ferroportin expression as shown by western blot analysis **(B)** left panel. After Omniscan treatment, hepcidin expression was decreased in comparison to untreated cells **(A)** lower panels, (**B)** right panels, western blot. Omniscan treatment with deferiprone increased the expression slightly. Representative blots from 3 separate experiments are shown. Values are means ±SD, obtained from 3 separate experiments, Significance of the data was determined by ANOVA, followed by paired-group comparisons. **p <0.01, compared with control, *p <0.05 (for hepcidin), **p <0.01 (for ferroportin) deferiprone- and Omniscan-treated compared with Omniscan alone. (Scale bars 100 μm for all).

We performed immunofluorescence staining of Omniscan and deferiprone-treated PBMC. As shown in Figs 13A and 14A, treatment of PBMC with Omniscan led to higher expression of CD163, CD34, procollagen-1, and prolyl 4-hydroxylase. Moreover, preincubation of PBMC with 25 μM of deferiprone for 24 hours inhibited most of the Omniscan-induced fibrotic markers. Expression of the iron regulator protein ferroportin was increased in Omniscan-treated cells, as detected by immunostaining (Fig 15A, upper panels). Deferiprone treatment markedly decreased Omniscan-induced, ferroportin-expressing cells. Omniscan treatment reduced hepcidin expression by fibrocyte-like cells, and deferiprone pretreatment partially ameliorated Omniscan-induced reduction in hepcidin expression (Fig 15A, lower panels). Western blot analysis further confirmed our immunostaining results (Figs 13–15, B panels). Panel C of Figs 13–15 demonstrate the quantitative data for western blot analysis.

## Discussion

Our studies indicate a novel role of iron in the pathophysiology of NSF in mice with CKD. We describe a mouse model with dermal changes characteristic of NSF and demonstrate in this model an accumulation of ferroportin-expressing fibrocyte-like cells and iron. We provide evidence for the important role of iron by demonstrating the beneficial effect of the iron chelator deferiprone. Our *in vitro* studies provide supporting evidence that Omniscan causes release of catalytic iron and that iron chelation with deferiprone inhibits Omniscan-induced differentiation of PBMC into ferroportin-expressing fibrocyte-like cells.

We have previously observed that gadolinium contrast administration in patients with CKD induces iron mobilization that precedes the eventual development of overt NSF [5]. Further, we and others have demonstrated that iron accumulates in the tissues and fibrocytes of patients with NSF [3,14,15]. Iron metabolism is tightly regulated in the body and excess iron is stored in macrophages. The body sequesters iron to limit iron-mediated oxidative injury through expression of hepcidin, an innate antimicrobial peptide synthesized by the liver. Hepcidin induces iron sequestration through down-regulation of the iron-exporting protein, ferroportin [16]. Thus, conditions that decrease hepcidin (such as hereditary hemochromatosis) are associated with increased ferroportin-mediated iron export and tissue-iron accumulation. Further, ferroportin expression in macrophages regulates the inflammatory role of macrophages and their potential pro-injury role. Thus, increased ferroportin expression would result not only in iron-mediated injury but also a macrophage-driven pro-inflammatory response [17,18]. We have recently demonstrated that gadolinium contrast induces differentiation of PBMC into ferroportin-expressing cells and that there is increased infiltration of these cells in the tissues of patients with NSF [6]. Gadolinium-containing compounds are known to induce metal transcription factor-1 (MTF-1), a known regulator of ferroportin expression. MTF-1 and ferroportin are critical for transitional metal efflux from the cells. Thus, we speculate that gadolinium chelates induce ferroportin in an MTF-1 dependent manner to facilitate gadolinium efflux but at the cost of perturbing iron homeostasis. In the current study, we provide evidence for the importance of iron by demonstrating that iron chelation using deferiprone partially restores monocyte hepcidin expression, prevents in vitro differentiation of PBMC into ferroportin-expressing cells, and markedly attenuates tissue accumulation of ferroportin-expressing cells in NSF. Most importantly, this was associated with significant amelioration of Omniscan-induced NSF-like skin changes. Collectively, our findings establish the importance of iron in the pathogenesis of NSF.

While these findings are highly suggestive of the importance of iron in NSF, there are several limitations to our study. First, while animal models of NSF are useful to examine the pathophysiology of Omniscan toxicity, animal models may not completely recapitulate the natural course of NSF in humans [19,20]. Second, while deferiprone is a highly selective iron chelator, additional benefit due to chelation of free gadolinium could not be completely excluded. Lastly, while we have demonstrated that ferroportin-expressing cells are substantially reduced by iron chelation therapy and that this coincides with histologic protection against Omniscan toxicity, our studies do not directly establish the primary role of ferroportin-mediated iron export. Future studies with hepcidin therapy would be required to address this question.

In summary, we present evidence that iron participates in Omniscan-induced differentiation of PBMC into ferroportin-expressing cells, and that chelation of iron with deferiprone therapy is effective in preventing Omniscan-induced NSF-like skin changes in mice. Our findings open the possibility of using deferiprone pretreatment to prevent gadolinium toxicity and NSF in patients with advanced chronic kidney disease.
